# Mediating role of splitting in relation to attachment styles and shopping addiction

**DOI:** 10.3389/fpsyg.2023.1249591

**Published:** 2023-09-27

**Authors:** Sarah Allahvirdie Rezaieh, Nima Ghorbani, Hojjatollah Farahani

**Affiliations:** ^1^Department of Clinical Psychology, University of Tehran, Tehran, Iran; ^2^Department of Psychology, Tarbiat Modares University, Tehran, Iran

**Keywords:** attachment styles, addictive behavior, splitting, maladaptive behavior, compulsive consumption, shopping addiction

## Abstract

**Introduction:**

Shopping can provide a sense of satisfaction and pleasure; however, if a person’s excessive involvement in this behavior starts to negatively impact other aspects of their life, similar to other addictive behaviors like excessive internet use, gaming, and gambling, it may be classified as pathological. Given the lack of agreement regarding the classification of excessive shopping tendencies as a separate mental health condition or addictive behavior, taking a socio-emotional approach to explore the psychological factors that precede this behavior, may offer a better comprehension of it.

**Methods:**

The purpose of this study was to examine the relationship between attachment styles and excessive shopping behavior, as well as to investigate the potential mediating effect of defense mechanisms like splitting on this relationship. Using convenience sampling, a group of 457 stock market employees (116 female, 341 male) between the ages of 24 and 60 were recruited. The researchers utilized a set of validated psychological questionnaires to assess the employees attachment styles, shopping addiction, and splitting tendencies.

**Results:**

The results obtained from both the mediation model and path analysis suggest that attachment styles do not have a direct relationship with shopping addiction. Nonetheless, the study supports the impact of insecure anxious and avoidan attachment styles on splitting. Furthermore, the findings confirm that splitting has a mediating effect on the relationship between attachment styles and splitting.

**Discussion:**

The present study enhanced our comprehension of the subconscious mechanisms underlying shopping tendencies. Specifically, the findings suggest that excessive tendencies towards shopping can be considered a maladaptive response resulting from insecure attachment styles and the unconscious utilization of the splitting defense mechanism.

## 1. Introduction

Some individuals may not exhibit resilience when confronted with various types of hardship and discord because they may not have acquired effective coping responses within the context of their family and society. Some individuals may resort to maladaptive behaviors like addiction as a way of seeking comfort and relieving negative emotions, due to their inability to handle failure, insufficient coping mechanisms, and tendency to swiftly give up on challenging or unfavorable situations (Washton and Boundy, 1990, p. 63). Addiction is a chronic medical condition requiring treatment, as emphasized by the American Society of Addiction Medicine (ASAM) (2020), and its’ development is influenced by a complex interplay between brain circuits, genetics, environment, and life experiences. The impact of “the environment” and “life experiences” on addiction, as indicated by ASAM, highlights the significance of contextual factors in susceptibility to addiction (Kettleborough, 2021).

Addiction is characterized as a phenomenon in which a behavior brings pleasure and also helps to alleviate negative internal states. Despite adverse consequences resulting from this behavior, attempts to control it fail, and the behavior persists (Goodman, 1990). Individuals with addiction prioritize the immediate gratification of engaging in addictive behavior over their personal and professional obligations, leading to a lack of control over their behavior (Montourcy et al., 2018). Jacobs (1988), in his general theory model of addiction, proposed that addiction develops gradually in response to the need for stress relief. It is a complex and often unconscious process that involves an avoidance-escape mechanism. One possible explanation is that when experiencing distress, individuals may be more likely to seek immediate solutions, such as impulsive behaviors, to escape the unpleasant situation (Tice et al., 2001); as a result, the priority lies in enhancing one’s mood rather than monitoring and managing one’s conduct (Leith and Baumeister, 1996). It is evident that not all people turn to chemical addiction to escape from unpleasant emotions, some individuals may turn to alternative strategies and behaviors. The addition of behavioral addiction (rather than substance addiction) to the fifth edition of the Diagnostic and Statistical Manual of Mental Disorders (DSM) by the American Psychiatric Association (APA) in 2013 and the eleventh revision of the International Classification of Diseases (ICD) by the World Health Organization (WHO) (2019), marks a significant shift from the belief that addiction is primarily related to drugs and chemicals like cocaine, alcohol, and tobacco. This shift, therefore, included behaviors like gaming, pornography, sex, and social media as addictive (Alavi et al., 2012); however, there is still debate regarding behaviors like “shopping” as addictive.

Shopping is an activity that has a significant role in people’s daily lives and can be seen as a way to improve one’s mood. Hirschman and Holbrook (1982) pointed out that shopping serves both practical and social purposes. It is not just a mundane task, but also an enjoyable opportunity to socialize and have fun, and Solomon et al. (2006), suggested that people use shopping as a way to relax, socialize, scape boredom of daily routines or cope with negative emotions (Kirezli and Arslan, 2019; Kettleborough, 2021). However, shopping can be considered pathological or addictive if done excessively, and this notion has led researchers to entitle the excessive urges to buy-shop with diverse terms such as compulsive buying, impulsive buying, or buying addiction. This disagreement makes it challenging to establish a clear definition for excessive shopping urges in view of the fact that behaviors such as shopping, using mobile phones, playing games, or studying, are our everyday life routines, even done excessively (Sussman et al., 2011), and there is a growing concern to avoid pathologizing common behaviors (Kardefelt-Winther et al., 2017). Thus, there is a need to understand the underlying psychological process of behaviors labelled as addictive.

The existing literature refer to various dynamics, while studying excessive shopping tendencies. For instance, Kyrios et al. (2004) referred to the self-worth improvement or reduction of negative mood as the desired expectation of compulsive buyers. They also found moderate link between compulsive buying and depression symptoms. Another study indicated that shopping episodes pushed by low self-control and emotional difficulties, such as anxiety and depression, further provoke strong urges to compulsive shopping (Bechara, 2005). Müller et al. (2010) also proposed that compulsive shopping coincides with other mental health conditions such as mood and anxiety disorders, impulse control disorders, and eating disorders. Arda and Andriany (2019), on the other hand, proposed that individuals with unmet intimate relationship expectations have more inclinations towards impulsive shopping. Other studies (Topino et al., 2022) indicate that addictions can be noticed in a relational context like attachment and therefore proposed that this condition is likely an attachment disorder (Flores, 2004; Schimmenti et al., 2012) so that insecurity in the attachment system leads affected individuals to be susceptible (Gori et al., 2022) in developing behavioral addictions (Estévez et al., 2017) including excessive shopping (Ünübol et al., 2022). Therefore, one of the main theories in research on emotional dysregulation, mood disorders, and addiction, including shopping addiction, is the attachment theory (De Rick et al., 2009; Norris et al., 2012; Fletcher et al., 2014; Norberg et al., 2018; Kettleborough, 2021); however, to better understand the pathways to excessive shopping, more studies are required to investigate the underlying mechanisms affected by insecure attachment styles that would lead to strong urges to shop.

Although a growing body of evidence highlighted the role of the attachment system in the development of behavioral addiction, including compulsive shopping, little attention has been paid to the role of defensive mechanisms such as splitting as a mediator between attachment style and excessive shopping. Therefore, the first aim of the current study was to investigate the underlying psychological process, such as attachment styles and defense mechanism of splitting, that may contribute to excessive shopping in the Iranian sample. Moreover, most research in the field targeted students (high school and university) (Müller et al., 2013; Duroy et al., 2014), the general population (Otero-López and Villardefrancos, 2014; Maraz et al., 2016) and shopping mall customers (Ridgway et al., 2008; Lejoyeux et al., 2012; Maraz et al., 2016), and the stock market employees have not been studied, despite spending a considerable amount of time buying and selling on trading platforms. These traders also devote time to shopping for their daily necessities, making them ideal candidates for obtaining richer data; therefore, to gain a deeper understanding of the propensity towards excessive shopping, we recruited stock market employees in this study as our second aim. According to our hypothetical model, attachment insecurities, such as anxious and avoidant insecure attachment styles, were positively associated with excessive shopping. In addition, the defense mechanism of splitting mediated the relationship between attachment styles and excessive shopping urges.

### 1.1. Shopping addiction

Researchers of consumer behavior, O’Guinn and Faber (1989), introduced the term “compulsive consumption” to describe the pattern of repetitive, consistent, and chronic buying behavior in response to negative incidents or emotions, or as a means of relieving stress, anxiety, depression, or boredom. Different terms have been used to describe the strong urge to shop excessively, such as “compulsive shopping” according to Black (2007). Other writers have referred to it as “pathological buying” (Müller et al., 2015), and “addictive disorder” (Hartston, 2012). Trotzke et al. (2020), proposed that there exist phenomenological parallels between buying-shopping disorder and behavioral addictions, and Müller et al. (2021) classified Buying-shopping disorder as a behavioral addiction. Researchers have also linked compulsive shopping to “affective disorder” (Lejoyeux et al., 1995; Lejoyeux and Weinstein, 2010) and “mood disorder” (Kesebir et al., 2012). Recent studies have proposed the term “behavioral addiction” (Piquet-Pessôa et al., 2014; Andreassen et al., 2015; Mestre-Bach et al., 2017; Kyrios et al., 2018; Potenza et al., 2018; Müller et al., 2019) to describe this strong urge to buy or shop. Despite being recognized by researchers as a potential behavioral addiction, the latest version of the DSM (DSM-5TR, 2022) has not yet classified shopping addiction as an official mental health condition or a mental disorder. Additionally, the 11th edition of the International Classification of Diseases (ICD-11, 2019) has categorized shopping addiction as an example of other specific impulse control disorders. One of the potential reasons for this issue might be lack of agreement among researchers regarding the factors that contribute to excessive shopping. For example, some studies have suggested that anxiety (Williams, 2012a), materialism and identity issues (Claes et al., 2016; Ahi et al., 2019), depression (Faber and Christenson, 1996; Müller et al., 2010), impulsivity (Billieux et al., 2008; Black et al., 2012; Williams, 2012b), narcissism and perfectionism (Rose, 2007), hedonism, escapism and negative mood reduction (Kirezli and Arslan, 2019), social pressures, negative emotions, addiction to the internet, and psychological instability such as depression, anxiety, low self-esteem, and feelings of loneliness (Suresh and Biswas, 2019) may all contribute to excessive shopping. Müller et al. (2015) suggested that buying and shopping behaviors are primarily used as a means to regulate negative emotions, attain pleasure, or cope with self-inconsistency. Other studies have proposed that non-substance-related addictions serve as coping mechanisms and avoidance strategies (Vala, 2016) to deal with emotional distress. They also emphasized the crucial role of attachment styles in the development of such addictions (Estévez et al., 2019). Davidson and Ireland (2009) highlighted the importance of secure bonds with attachment figures stating that individuals with insecure attachment lack both insufficient skills to maintain social relationships and do not have a supportive social network, which would be the basis of their inclination towards addictive behaviors as a coping response or a substitute for their lack (Estévez et al., 2019). These studies hypothesized that insecure attachment, particularly anxious attachment, and emotion dysregulation are significant psychological characteristics that increase the likelihood of engaging in addictive behaviors such as alcohol, marijuana, and texting (Liese et al., 2020). Monacis et al. (2017a,b) found that behavioral addictions such as problematic internet use, gaming addiction, and social media addiction were negatively correlated with secure attachment. People with insecure attachment may have less ability to self-regulate during stressful situations, resulting in the search for external methods to reduce stress (Nakhoul et al., 2020).

Taking an alternative approach, one might consider repetitive and compulsive behaviors as an insufficient coping response for dealing with emotions, as proposed by Fairbairn et al. (2018); Therefore, it is crucial to examine the excessive shopping behavior, commonly referred to as a behavioral addiction, from a socio-emotional viewpoint, and explore the underlying mechanisms that contribute to inadequate emotional regulation.

### 1.2. Attachment

According to Bowlby (1969), attachment styles play a crucial role in socio-emotional development. The quality of interactions between parents and children can profoundly influence a child’s mental representations of themselves and others, known as “working models.” These models serve as the foundation for a person’s social experiences and perceptions of their environment, leading to individual differences in attachment organization (Bowlby, 1969, 1973, 1980; Main et al., 1985), which in turn affects emotion regulation and the representation of self and others (Kobak and Sceery, 1988). Therefore, working models can be a source of vulnerability if a person has a history of insecure relationships, while secure relationships can promote resilience (Ainsworth and Bowlby, 1991). An impaired sense of attachment security, such as the presence of secondary attachment strategies, namely anxious and avoidant attachment (Main, 1990), can be considered a risk factor for emotional issues and psychopathology (Mikulincer et al., 2003; Mikulincer and Shaver, 2016). The attachment system is proposed to serve as a form of affect regulation and a motivational system (Velotti et al., 2021) that develops during an individual’s early years and is considered one of the influential factors in emotion regulation and adaptive coping (Lazarus and Folkman, 1984). It assists individuals in learning how to manage and regulate negative emotions while under distress (Gross, 2002; Mikulincer et al., 2009).

Traumatic and adverse experiences involving attachment figures can lead to distorted, distinct, and unrelated mental representations of oneself and others. Such experiences may trigger the emergence of repetitive thoughts and exaggerated concerns regarding one’s self-worth or image (Ghorbani, 2016), resulting in difficulties regulating emotions and limited access to effective responses; therefore, individuals who have difficulty regulating their emotions may resort to risky and maladaptive behaviors as a means of coping (Oshri et al., 2015; Mirhashem et al., 2017; Espeleta et al., 2018). Topino et al. (2022) highlighted that insecure attachment is a risk factor for alcohol and substance abuse (Iglesias et al., 2014; Zakhour et al., 2020), as well as addictive behaviors, such as Internet and technology (Kim and Koh, 2018; Sun and Zhang, 2021), and contrary to insecure attachment styles, secure attachment protect individuals against addictions (Monacis et al., 2017a,b), and psychopathological disorders (Armour et al., 2011; Ensink et al., 2021). According to Vaillant (1998, pp. 166–167), individuals with attachment injuries have a greater propensity for drugs and alcohol, and Kohut (1978, p. 225) suggests that drug addiction results from a disturbance in attachment formation. Additionally, research has found that ambivalent and avoidant attachment styles positively and significantly predict non-substance-related addictions such as gambling disorder, video game addiction, and problematic internet use (Estévez et al., 2017; Liu et al., 2020). Norris et al. (2012) found that material possessions are a substitution for social connection as individuals with anxious attachment styles have difficulties maintaining close relationships. In addition, Norberg et al. (2018) proposed that people with insecure attachment have concerns with personal ties and, therefore, seek comfort in buying and collecting objects. Topino et al. (2022) also found that secure attachment negatively correlates to compulsive online shopping and fearful attachment is positively related to compulsive online shopping. Hence, attachment theory and attachment orientations are critical in comprehending how individuals experience and regulate their emotions (Mikulincer and Shaver, 2018); failure to develop emotional regulation skills within the career-child relationship due to the insecurity in attachment styles can severely compromise an individual’s ability to effectively manage stressful situations, which, in turn, significantly increases the likelihood of relying on immature and/or neurotic defense mechanisms during periods of stress (Besharat and Khajavi, 2013). In a dysfunctional way, immature defenses protect an individual from an internal state of fear that they perceive as threatening (Freeman et al., 2002; Marrazzo et al., 2016; Ciocca et al., 2017). As a result, individuals with disrupted attachment systems and immature defenses may struggle with developing effective regulatory strategies, which can lead to maladaptive responses. Lack of effective coping responses during times of intense emotion may turn to impulsive behaviors as a seemingly effective but short-term method of managing distress (Espeleta et al., 2018).

### 1.3. Splitting

Psychological defenses are typically involuntary patterns of emotions, thoughts, or behaviors that help individuals cope with challenging situations. These coping processes are often referred to as “unconscious” (Cramer, 2015; Vaillant, 2020) and were first discussed in psychoanalytic literature by Freud (1894) and further explored by Anna Freud in 1937 (Richardson et al., 2022). Defense mechanisms are crucial processes that automatically regulate the self and effectively reduce cognitive discrepancies. They play a significant role in minimizing sudden changes in external and internal reality by distorting the perception of threatening events (Vaillant, 1994). Defense mechanism involves changing one’s mental representations based on an unconscious desire which helps to prevent potential suffering (Miceli and Castelfranchi, 2001). According to Laczkovics et al. (2020), one’s attachment style is a crucial factor in determining the defense mechanisms that are exhibited (Richardson et al., 2022). Defenses are designed to regulate the attachment system and alleviate distressing emotions associated with negative expectations at both the intrapersonal and interpersonal levels, and are closely linked to emotion regulation (Malik et al., 2015; Kobak and Bosmans, 2019). Ciocca et al. (2020) proposed that defense mechanisms are the mediators in the relationship between attachment styles and psychological and/or behavioral implications. According to the preoccupied attachment model proposed by Griffin and Bartholomew (1994), individuals with high attachment-anxiety who hold a negative self-image and positive other-image, tend to rely on splitting-self and splitting-other mechanism. The defense mechanism of splitting, rooted in attachment theory (Bowlby, 1969), is employed to manage high levels of ambivalence and relationship failures (Kernberg, 1984). This defense mechanism, which is often used interchangeably with dissociation, is considered a primitive defense in psychoanalytic literature (Ghorbani, 2016). Kernberg (1975) has characterized splitting as a cognitive process that occurs during early childhood and involves the failure to integrate positive and negative self-images and images of significant others. In normal development, contradictory emotions and experiences are integrated into a cohesive set, allowing individuals to experience a mix of emotions towards others. However, in abnormal development, this capacity for emotional cohesion and ambivalence does not emerge, and splitting becomes a dominant psychological defense (Gerson, 1984). Individuals who use splitting separate and disconnect mental images of themselves and others to avoid anxiety and ambivalence because they lack the psychological capacity to tolerate conflicts (Ghorbani, 2016). The primary aim of splitting as a defense mechanism is to maintain psychological stability and coherence, but paradoxically, it leads to instability and fluctuations in one’s experiences, causing mood swings and difficulties in interpersonal relationships (Gould et al., 1996). Rather than acknowledging conflict and ambivalence, individuals who use splitting often retreat into a fantasy world (Ghorbani, 2016). Lopez et al. (1997) postulates that normal functioning individuals with impaired early bonds with caregivers are more susceptible to relying on splitting defense mechanisms to manage internal tension. Gould et al. (1996) found that splitting is positively correlated with depression, negative emotion, and low self-esteem, and negatively correlated with positive emotion. Put differently, splitting can lead to emotional dysregulation, which has been identified as a possible predictor of both chemical and behavioral addiction in various studies (Petit et al., 2015; Estévez et al., 2017; Poole et al., 2017). It is noteworthy literature is scarce regarding the relationship between the defense mechanism of splitting and addictive behaviors.

Addictive behaviors may serve as maladaptive strategies for regulating emotions (Fairburn et al., 2018); therefore, the aim of this study is to investigate whether there is a link between insecure attachment styles and excessive shopping behavior, and if this relationship is mediated by the defense mechanism of splitting. The study aims to explore whether individuals with insecure attachment styles are more prone to engage in splitting, leading to excessive shopping tendencies as a maladaptive coping response. As far as we know, this research is the first of its kind to investigate the mediating role of splitting in the relationship between attachment styles and excessive shopping within the stock market community in Iran, as no prior research has been conducted on this topic in the country.

### 1.4. Hypothesis

Our initial hypothesis posits that a weak bond between a child and caregiver may lead to a higher inclination towards excessive shopping (H1). According to existing literature, attachment denotes the nature of the relationship between a parent and child and plays a crucial role in socio-emotional growth. Consequently, disruptions in the attachment system could increase the risk of developing behavioral addiction.

We also hypothesize that there exists a direct correlation between the splitting mechanism and excessive shopping (H2).

**Figure 1:**
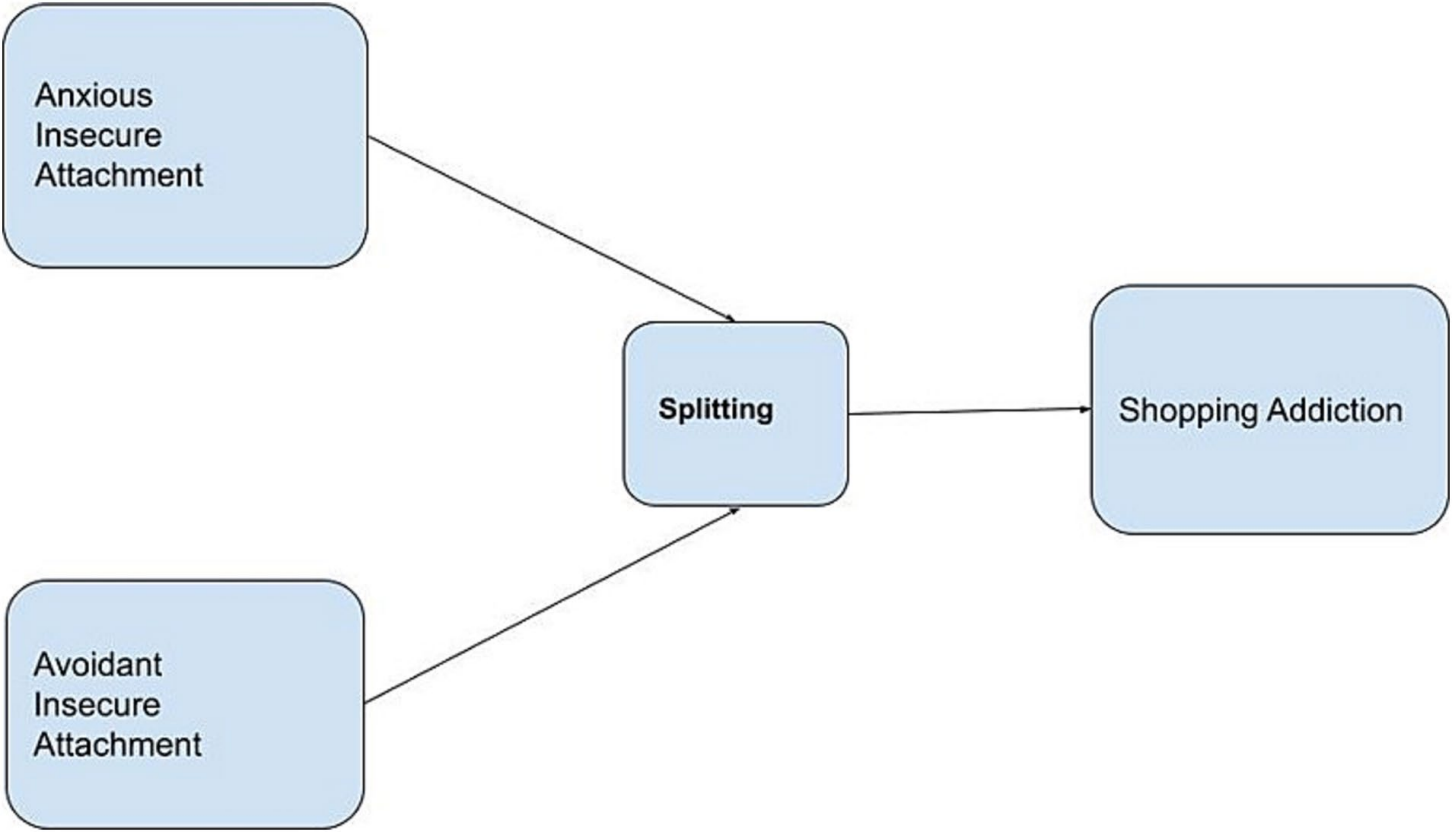
Theoretical model.

We propose a third hypothesis that suggests the defense mechanism of splitting fully or partially mediates the relationship between insecure attachment styles and excessive shopping behavior (H3), as demonstrated in Figure 1.

## 2. Materials and methods

### 2.1. Participants

Four hundred fifty-seven stock market employees [M = 74.6% (341); *F* = 25.4% (116)], working in Tehran’s stock market, aged between 24 and 60, took part in the study. As regards marital status, 187 (40.9%) were unmarried, 263 (57.6%) were married or living with a partner, and 7(1.5%) were divorced or separated. Thirty-nine participants (8.5%) had a high school diploma, 168 (36.8%) had graduated in the first cycle degree, 200 (43.8%) had graduated in the second cycle degree, and 20 (10.9%) had a Ph.D. Most of the participants were male (75.6%), married (57.6%) and graduated in the second cycle degree (43.8%). The inclusion criteria for this research were that the participants must have been employees or active in the stock market and were in the age range of 24–60; those who did not meet the inclusion criteria were excluded from the study. This study targeted a non-clinical population.

### 2.2. Procedure

A convenience sampling method was employed for the project. To conduct the survey, an online questionnaire was developed using Porsline software.^1^ The survey link,^2^ along with a set of questionnaires, was advertised on stock market channels and educational groups on Telegram. Stock market company employees and dealers participated voluntarily in the research. The survey did not collect any personally identifying information. Prior to data collection, the participants’ consent was obtained (electronically) and they were informed that the provided responses will be kept confidential and will not be disclosed elsewhere in accordance with ethical standards and principles of confidentiality and privacy, except for the purpose of research.

### 2.3. Measures

The first part of questionnaire included demographical information such as sex, age, marital status, and education level.

### 2.4. Attachment styles

The Revised Adult Attachment Scale (RAAS), created by Collins (1996), is an 18-question survey that evaluates attachment styles. Respondents rate their responses on a 5-point Likert scale, ranging from complete disagreement (1) to complete agreement (5). The questionnaire is comprised of three components: Anxiety, which measures anxiety in relationships, such as fear of abandonment or being despised; Dependence, which assesses a person’s trust in others and their availability; and Closeness, which evaluates discomfort in closeness and intimacy. A study conducted by Nikoogoftar in 2013 in Iran reported Cronbach’s α coefficients of 0.45 for the Dependence subscale, 0.52 for Closeness, and 0.75 for Anxiety. The study also utilized confirmatory factor analysis to evaluate the validity of the scale, and the results validated the existence of three attachment factors: secure (intimacy), avoidant (anti-dependency), and ambivalent (anxiety). Additionally, the study reported Cronbach’s α values of 0.22, 0.76, and 0.64 for the three subscales of secure attachment, anxious insecure attachment, and avoidant insecure attachment, respectively.

### 2.5. Online and in store shopping addiction

The Online Shopping Addiction Scale was utilized alongside the Bergen Shopping Addiction Scale to assess individuals’ inclinations towards both online and in-store shopping, as some people may not be inclined towards in-store shopping and instead prefer online purchases.

The scale comprises seven statements on a 5-point Likert scale ranging from complete disagreement (0) to complete agreement (4). The total score ranges from 0 to 28, with higher scores indicating greater levels of addiction to shopping. Although the validity and reliability of Bergen’s shopping addiction scale (2015) have yet to be evaluated in Iran, the research employed the translation-back-translation method. The Cronbach’s α coefficient in this study was 0.85.

The degree of addiction to online shopping was assessed using the Online Shopping Addiction Scale (OSAS), which comprises 18 statements rated on a 5-point Likert scale ranging from complete disagreement (1) to complete agreement (5). Scores on this scale ranged from 1 to 90, with higher scores indicating greater levels of addiction. Bagheri Sheykhangafshe et al. (2023) evaluated the validity and reliability of this scale. This study reported a Cronbach’s α coefficient of 0.93 to assess the scale’s internal consistency.

### 2.6. Splitting index

The Splitting Index was used to measure splitting, with a questionnaire consisting of 24 statements rated on a 5-point Likert scale ranging from complete disagreement (1) to complete agreement (5). The statements were categorized into three factors—self, family, and others—to assess splitting of images. Scores ranged from 24 to 120, with higher scores indicating higher levels of splitting. Unfortunately, there is no research available in Iran to validate the reliability of this scale. Therefore, a translated version of the available scale was used in the research. In this study, the estimated Cronbach’s α for the splitting scale was 0.83, and the Cronbach’s α for self, family, and others images were 0.76, 0.73, and 0.66, respectively.

### 2.7. Analytical procedures

In the current research, for the statistical analysis of the data, initially, the demographic indicators of the research subjects were presented with SPSS 21 software, and then the descriptive indicators of the mean, standard deviation, skewness, and kurtosis of the research variables were displayed. Subsequently, for the inferential analysis of the data, the correlation matrix of the research variables was presented, and structural equation modeling with Amos 24 software was utilized to determine the fit indices of the model with the data and to determine the direct and indirect effects of exogenous variables on endogenous variables. Finally, the bootstrap method with 2,000 sampling processes and a corrected 95% confidence interval was used to determine the mediating effects. Structural equation modeling is a robust multivariate method commonly employed in scientific research to assess direct and indirect effects on pre-assumed causal relationships.

## 3. Results

The normality and non-collinearity assumptions were verified prior to analyzing the data. To confirm the normality assumption for all variables, descriptive statistical analysis such as skewness and kurtosis indices were employed. The outcomes of this analysis are presented below: anxious attachment style (Ku = −0.456, Sk = −0.069), avoidant attachment style (Ku = −0.388, Sk = −0.128), secure attachment style (Ku = 0.087, Sk = −0.312), splitting from self-image (Ku = −0.569, Sk = −0.0124), splitting from family-image (Ku = −0.737, Sk = 0.463), splitting from others-image (Ku = − 0.154, Sk = − 0.356), online shopping addiction (Ku = − 0.086, Sk = 0.700) and in-store shopping addiction (Ku = − 0.332, Sk = 0.742). Chu and Bentler (1995) suggest that a skewness value of ±3 is a suitable cutoff point. In multivariate research, values above ±10 for the kurtosis index are usually problematic (Kline, 2015). The skewness and kurtosis values obtained for all variables indicate that the normality assumption is met. Table 1 presents the central tendency, dispersion indices, and correlation of research variables.

**Table 1:** Descriptive indices and correlation matrix of research variables.

Variable	M	SD	1	2	3	4	5	6	7	8
Anxious attachment	9.50	4.57	1							
Secure attachment	9.27	2.20	0.30**	1						
Avoidant attachment	8.18	3.53	0.56**	0.14**	1					
Splitting self-image	21.91	5.66	0.55**	0.10*	0.44**	1				
Splitting family-image	14.67	4.55	0.31**	0.01	0.30**	0.43**	1			
Splitting other-image	15.85	3.25	0.34**	0.06	0.30**	0.39**	0.36**	1		
Online shopping addiction	36.02	13.25	0.24**	0.05	0.18**	0.25**	0.28**	0.26**	1	
In-store shopping addiction	5.94	5.33	0.33**	0.09*	0.20**	0.29**	0.28**	0.29**	0.65**	1

**p < 0.01; *p < 0.05.

When correlation coefficients between variables exceed 0.85, multiple collinearities become an issue, leading to inaccurate model estimation (Kline, 2015). To address this, the correlation matrix between observed variables was examined, revealing the absence of multiple collinearities. The assumption of non-collinearity was further confirmed by assessing the Variance Inflation Factor (VIF) and Tolerance Index statistics. Specifically, all tolerance index values were greater than 0.10, and all variance inflation factor values were less than 10, indicating that non-collinearity can be assumed with confidence.

The study employed structural equation modeling to examine both direct and indirect effects. The research assumed a model where the splitting variable was included as a latent variable and comprised three components: splitting from self-image, family-image, and others-image. These components were used to measure the splitting variable. Additionally, the shopping addiction variable was also included as a latent variable, and two variables, namely online shopping addiction and in-store shopping addiction, were used to measure it. Lastly, the model included three observed variables, namely secure, anxious, and avoidant attachment styles.

**Figure 2:**
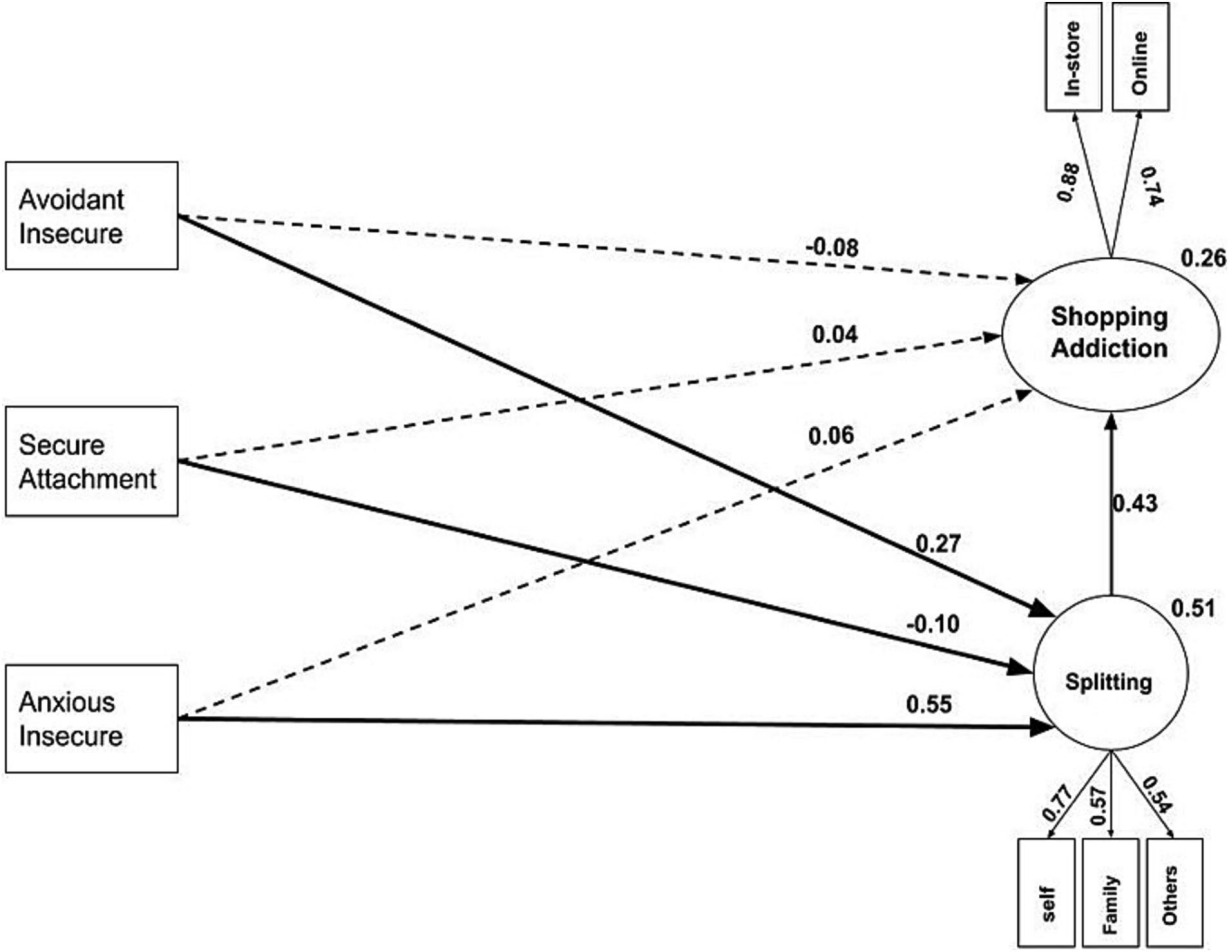
Standard path coefficients of research variables in the main model. Bold, continuous lines indicate paths of key interest and dashed lines represents non-significant paths.

Figure 2 and Tables 2, 3 depict the assumed model and demonstrate the direct and indirect effects, respectively.

**Table 2:** Structural model fit indices.

Fit indices	Cut off criteria	Hypothetical model
Chi-square (χ^2^)	–	26.865
The ratio of Chi-square to the degree of freedom	Values of 3 and less	2.067
Comparative Fit Index (CFI)	Values larger than 0.90	0.985
Incremental Fit Index (IFI)	Values larger than 0.90	0.986
Goodness of Fit (GFI)	Values larger than 0.90	0.985
RMSEA	Values smaller than 0.08	0.048
SRMR	Values smaller than 0.08	0.029

**Table 3:** Examining the direct relationships of variables in the research model.

Predictor variable	Criterion variable	Unstandardized coefficients	Standardized coefficients	Standard error	*t*	*p*
Anxious attachment style	Shopping addiction	0.05	0.05	0.08	0.69	0.48
Secure attachment style	Shopping addiction	0.08	0.03	0.11	0.73	0.46
Avoidant attachment style	Shopping addiction	−0.11	−0.08	0.08	−1.3	0.19
Splitting	Shopping addiction	0.54	0.50	0.11	4.75	0.001
Anxious attachment style	Splitting	0.52	0.55	0.05	9.34	0.001
Secure attachment style	Splitting	−0.19	−0.09	0.09	−2.03	0.04
Avoidant attachment style	Splitting	0.33	0.27	0.06	4.93	0.001

In Figure 2 bold, continuous lines indicate paths of key interest and dashed lines represents non-significant paths.

The fit indices of the structural model, as presented in Table 2, confirm that the hypothetical model used in the research is well-supported, indicating a good fit for the model. An illustration of a good model fit can be seen from the fit indices of CFI, IFI, and GFI in the current model, which were 0.985, 0.986, and 0.985, respectively. This exceeds the standard threshold of 0.90, indicating that the model has a good fit. In conclusion, the RMSEA fit index, which is considered by some statisticians to be a crucial indicator of model fit, was 0.048. This value is lower than the threshold of 0.08, indicating a good fit of the model. Therefore, the research hypothesis that splitting mediates the relationship between attachment styles and shopping addiction is confirmed.

The direct and mediation effects of the research variables are illustrated in Tables 3, 4. These results enable confirmation or rejection of the direct and indirect impact of the research variables on shopping addiction.

**Table 4:** Indirect relationships of variables in the research model.

Predictor variable	Mediating variable	Criterion variable	Unstandardized coefficients	Lower limit	Upper limit	*p*
Anxious attachment style	Splitting	Shopping addiction	0.28	0.16	0.47	0.001
Secure attachment style	Splitting	Shopping addiction	−0.10	−0.27	−0.01	0.02
Avoidant attachment style	Splitting	Shopping addiction	0.18	0.09	0.32	0.001

If the *t* statistic falls outside the range of (+1.96/−1.96) or the significance level is below 0.05, it indicates a significant relationship between two variables. Table 3 indicates that there is no significant direct path from anxious attachment style to shopping addiction variable (*t* = 0.649, *β* = 0.058). The direct path of secure attachment style to shopping addiction is not significant (*t* = 0.737, *β* = 0.038); the direct path of avoidant attachment style to shopping addiction is not significant (*t* = −1.3, *β* = −0.084); but, the direct path of splitting to shopping addiction is significant (*t* = 4.754, *β* = 0.507); the direct path of anxious attachment style to splitting is significant (*t* = 9.346, *β* = 0.551); the direct path of the avoidant attachment style to the splitting is significant (*t* = 4.939, *β* = 0.271); and the direct path of secure attachment style to splitting is negatively significant (*t* = −2.038, *β* = −0.096).

The bootstrap method with a 2,000-times sampling process was utilized to assess the indirect effect. Table 4 shows that the secure attachment style has a statistically significant negative indirect effect on shopping addiction through splitting (*p* < 0.05, *b* = −0/104); Splitting mediates a significant indirect effect of the anxious attachment style on shopping addiction (*p* < 0.05, *b* = 0.287); and splitting plays a significant mediating role in the indirect effect of the avoidant attachment style on shopping addiction (*p* < 0.05, *b* = 0/183).

## 4. Discussion

The debate surrounding the classification of “excessive urges to shop” under an accurate definition is ongoing, and there is still no consensus. In this study, we aimed to examine underlying psychological factors affecting excessive shopping tendencies to learn whether attachment styles and the defense mechanism of splitting would impact the inclination to engage in excessive shopping; we used a mediation model to explore this relationship. As far as we know, this research has not previously been conducted in Iran. The findings showed that the direct relationship between anxious insecure attachment, avoidant insecure attachment, and secure attachment with shopping addiction is not significant. As a result, the first hypothesis (H1) has been rejected. The findings suggest that, an individual’s attachment style provides the basis for the behavioral and regulatory system (Estévez et al., 2019), but it does not necessarily directly lead to pathological behavior. Instead, this system influences other psychological structures like defense mechanism, impulse control, coping strategies, and emotion regulation. Attachment system defects decrease the capacity for self-regulation during stressful situations (Nakhoul et al., 2020), leading to inadequate adaptation of emotion regulation strategies (Liese et al., 2020) and employment of defensive cognitive processes to manage internal crises (Mikulincer and Orbach, 1995). In a review study by Subocz (2022), it was found that a child’s insecure attachment relationship inhibits the formation and development of neural connections essential for emotion regulation. As a result, the child may become more susceptible to future traumatic experiences (Schore, 2009) and more vulnerable to psychopathology. Insecurity in the child-career’s relationship would lead individuals to develop a negative self-concept, which causes negative beliefs about self-worth and being loved; in this case scenario, people likely avoid such feelings and concepts by adapting maladaptive or addictive behaviors (Pace et al., 2013). Therefore, the findings of this study, in line with previous research (Estévez et al., 2017), suggest that anxious and avoidant insecure attachment style may serve as predictors or predisposing factors for the development of behavioral addictions. In addition, the results indicate a significant direct relationship between splitting and shopping addiction, revealing that the splitting mechanism has a significant influence on the propensity towards shopping addiction (H2). Craparo et al. (2014) reported that addictive behaviors have a dissociative nature that allows individuals to regulate negative emotions. Splitting, akin to dissociation, is an inefficient psychological mechanism and self-regulatory strategy that aids individuals in coping with psychological distress. It involves separating and disconnecting heterogeneous mental images of oneself and others to avoid painful ambivalence (Ghorbani, 2016). While splitting attempts to maintain stability and coherence within the psyche, it paradoxically results in instability and fluctuation in one’s experience (Gould et al., 1996). Therefore, this defense mechanism may serve as a meta-diagnostic factor in the development of addictive behaviors (Grajewski and Dragan, 2020).

Earlier research has supported the notion that poor childhood bonding and experiences can indirectly contribute to addiction-related problems through emotion dysregulation, severity of dissociative experiences, and emotional disorder (Espeleta et al., 2018; Evren et al., 2019; Schaefer et al., 2021). This study aimed to examine the mediating effect of splitting mechanisms on the relationship between attachment styles and shopping addiction (H3). The results of the path analysis indicated that splitting has a significant mediating effect on the relationship between anxious insecure attachment and avoidant insecure attachment on shopping addiction. Moreover, splitting was found to negatively mediate the association between secure attachment style and shopping addiction. The present results are consistent with earlier research conducted by Besharat et al. (2001), McMahon et al. (2005) and Lopez et al. (1997), which exhibited defense mechanisms are affected by attachment styles and weak attachment bonds are associated with insecure attachment orientations and propensity for splitting during adulthood. Securely attached individuals experience higher levels of positive emotions due to their positive self-concept; therefore, they tend to rely more on mature defense mechanisms in distressing conditions. Conversely, people with insecure attachment hold a negative self-concept, which makes them experience higher levels of negative emotions, and predispose them to utilizing more immature and/or neurotic defense mechanisms (Feeney and Kirkpatrick, 1996; Besharat et al., 2001). Specifically, individuals with secure attachment tend to use more mature coping strategies, while those with anxious and avoidant insecure attachment styles are more prone to using splitting mechanisms. Individuals with insecure attachment styles lack internal stability, making them more vulnerable and inclined to use splitting as a coping mechanism when faced with distress (Lopez et al., 1997); as highlighted in a study by Besharat and Khajavi (2013) immature and neurotic defenses are activated to guard individuals with insecure attachment from unpleasant impact of negative self-image. However, this maladaptive mechanism leads to disruption in emotional processing, resulting in emotional dysregulation and potentially participating in shopping behavior as a secondary coping mechanism to divert attention from self-awareness and thoughts of previous injuries. Faber and Vohs (2004) attributed most compulsive purchases to consumers’ attempts to escape from self-awareness, suggesting that shopping becomes an “emotional distress reliever” and a way to deal with stress (Otero-López et al., 2021). Moreover, Krueger (1988) postulate that underlying factors in excessive shopping urges are instable self-image and a strong desire to feel alive. Therefore, it can be inferred that poor attachment bonds may lead to the use of immature defense mechanisms, such as splitting, which causes emotional dysregulation, and addictive behaviors such as shopping may result from this maladaptive way of regulating dysregulated emotions. As a result, the third hypothesis (H3), the mediation model, is confirmed.

Early secure bonds with caregivers equip individuals to internalize consistent, unified, and positive self and other images (Lopez et al., 1997). The attachment system is both a protective and risk factor (Black-Hughes and Stacy, 2013), mainly because this system exerts a direct effect on defense mechanisms, and depending on what attachment style people develop, it can affect the use of mature, neurotic, or immature defenses; as proposed by Monacis et al. (2017a,b) and Vally et al. (2020), insecure attachment styles and defense mechanisms make individuals prone to online gaming addiction, social media addiction and problematic internet use. Thus, within this frame, maladaptive behaviors and addictions of all kinds (chemical and behavioral) may be seen as a consequence of insecurity in the attachment system and simultaneously deal with distorted and incoherent self-images caused by insecure attachment styles (Hosseinifard and Kaviani, 2015).

### 4.1. Limitations and future direction

This research contributes to our understanding of the socio-emotional factors that drive excessive shopping tendencies, including attachment styles and defense mechanisms like splitting. However, there are certain limitations to this study. First, the research’s cross-sectional design, makes it impossible to establish causal relationships. Second, our findings have limited external validity due to the sampling procedure we employed, which involved a convenience sample sourced online. Therefore, our results cannot be generalized to the wider population. Third, the non-uniform age distribution among participants and the disproportionate number of male-to-female participants (with three times as many males as females) make it challenging to compare gender differences in tendencies towards excessive shopping. Despite previous literature suggesting a low male response rate and their reluctance to respond to inquiries about shopping (Dittmar, 2005), this study found that the majority of respondents were male. Fourth, this research is limited by its reliance on self-report data collection; the cultural significance of the role of parents, particularly mothers in Iranian culture, may have influenced respondents’ answers to questions related to attachment styles, which is particularly important to note in the attachment style questionnaire. Fifth, some of the findings were inconsistent with the existing literature that suggested secure attachment styles have a significant and negative relationship with behavioral addictions (Monacis et al., 2017a,b; Topino et al., 2022); in this study, no significant association between attachment and shopping addiction was found, which may be due in part to the small size of the Cronbach alpha of the tool. Therefore, given these results, researchers should be cautious in using this tool in the future. Sixth, there has been no investigation into other psychological mechanisms and constructs, such as coping skills, self-esteem, and perceived stress. Future research, need to address other psychological constructs, while investigating the shopping tendencies in non-clinical samples. In addition, future research, may exert psychologically relevant exclusion criteria such as Axis 1 or Axis 2 disorders. Finally, to enhance the comparability and generalizability of the findings, future research ought to concentrate on individuals who are actively seeking treatment or have been diagnosed with shopping addiction.

## 5. Conclusion

To summarize, the present study’s tested model has enhanced the comprehension of psychological processes that underlie excessive shopping behavior. Specifically, it illuminates how insecure attachment styles and the splitting mechanism contribute to shopping tendencies even in non-clinical sample. The results indicate that individuals with secure attachment styles who do not use splitting may have a protective factor against the risk of developing such dysfunctional behavior. In contrast, insecure attachment styles and splitting are risk factors for excessive shopping urges. Therefore, excessive shopping may seem like a deceptive and counterproductive remedy for attachment wounds; as proposed by Fletcher et al. (2014), early secure connections are of great importance, considering the impact of the attachment system on maladaptive and addictive behaviors. This study’s findings could have implications for clinical intervention. Pinpointing the fundamental mechanisms that stimulate the desire to make purchases is crucial for customizing addiction prevention programs.

## Data availability statement

The raw data supporting the conclusions of this article will be made available by the authors, without undue reservation.

## Ethics statement

APA ethical standards were followed in the conduct of the study.

## Author contributions

SAR collected the data and wrote the first draft of the manuscript. NG and HF provided critical revision of the manuscript and important intellectual contributions. All authors contributed to the article and approved the submitted version.

## Footnotes

^1^ https://survey.porsline.ir/

^2^ https://survey.porsline.ir/n/survey/473946/results/table/
